# Rates of heat, mass and momentum transfer in a magnetic nanofluid near cylindrical surface with velocity slip and convective heat transfer

**DOI:** 10.1016/j.heliyon.2024.e27675

**Published:** 2024-03-07

**Authors:** Tadesse Walelign

**Affiliations:** Department of Mathematics, Debre Tabor University, PO.Box 272, Ethiopia

**Keywords:** Transport rates, Nanofluids, Cylindrical solid, Optimal homotopy analysis method

## Abstract

The main attention of this study is to give analytic investigation on the behavior of a nanofluid transport rates in response to a continuous variation of parameters. After reducing the governing boundary layer equations in to a set of convenient ordinary differential forms, the efficient optimal homotopy analysis method has been successfully implemented to the set of nonlinear problems. In this analysis, it is found that significant variations of heat, mass and momentum transfer rates are identified with the changes in the values of magnetic field, porosity parameter and diffusion thermo effects. Among other things, the findings of this study will contribute for better understanding and predicting of fluid transport rates near cylindrical surfaces. This will help both theoretical scientists and practical engineers to estimate the degree to which various factors affect the quality of manufacturing products.

## 1 Introduction

Nearly all our day to day activities, manufacturing processes and utilization of technological devices involves fluid transport phenomena. On the other hand, advanced heating or cooling is one of the most important concerns in material processing and efficient utilization of technological devices. It determines the amount of energy or time it requires or quality of the end products in manufacturing industries. Also, the size, safety and efficiency of technological devices as well as their service time strongly depend on the amount of heat removed after it has been generated by the devices [1]. To this end, fluids play significant roles either to add or remove heat to or on a material surface. However, many of the naturally occurring fluids such as ethylene glycol and water a considerable thermal resistance [2], [3]. A number of studies have been conducted to enhance the thermal conductivity of the existing fluids. Some of the early investigations pointed out that addition of nanometer sized particles on to the naturally occurring fluids dramatically improves the thermal conductivity of the fluids with nanoparticles [4], [5], [6], [7], [8], [9], [10], [11], [12].

Zulkifli et al. [13] analyzed a revised model on a boundary layer flow of a Nanofluid over a moving plate in the presence of viscous dissipation using Runge-Kutta Felhberg numerical procedure. They reported that the temperature profile grows with the increase in viscous dissipation. Owhaib and Al-Kouz [14] analyzed the heat transfer of a nanofluid in a film flow by considering the effect of thermo-migration and haphazard motion of nanoparticle properties. Sreedevi et al. [15] studied the transfer of heat and mass in a nanofluid flow over stretching surface using the finite element method. Najib and Bachok [16] investigated the rate of heat transfer and skin friction in a viscous flow of fluid over a shrinking cylinder. They pointed out that temperature distribution is enhanced with the Brownian and thermophoresis effects. Jawad et al. [17] analyzed a bioconvection Darcy-Forchheimer flow of Casson nanofluid over a rotating disk with entropy generation. Upon implementation of homotopy analysis, they reported that the rate of entropy generation increases for greater values of the Brinkman number, Casson fluid parameters, and magnetic parameters.

The rate of heat transfer between a solid surface and a surrounding fluid can be quantified in terms of the so-called the Nusselt number. The rate of nanoparticle mass transport near the solid surface can be measured in the form of Sherwood number, which is an expression based on the gradient of nanoparticle concentration. Also, the rate of fluid momentum transfer in the interface can be predicted in terms of the local skin friction coefficient. Owing to the practical relevance of these quantities in predicting the rates of fluid transport phenomena, several studies have been conducted to explain their behavior in relation to various flow parameters. Unfortunately, majority of the reports are based on the discrete value of the parameters [18]. This does not give a full insight on the range of the parameters as well as the actual behavior of the quantities under consideration.

Due to the complex nature of fluid flow behavior and the limitation of mathematical methods, it is common to neglect relevant parameters from the governing equations. For instance, a greater number of studies [19], [20], [21], [22] did not consider curvature and angle of elevation of the stretching surface. Also, the influence of velocity slip parameter and cross diffusion effects are not considered in several studies [23].

Consequently, the present study explores the behavior of fluid transport rates in relation to the changing values of several parameters in continuous domains. Basically, the study employed the optimal homotopy analysis method, which is a powerful tool having the combined advantages of numerical and analytical methods [26], [27]. It is therefore believed that the findings of this study will give important insights for both theoretical and experimental scientists about the flow problem under consideration.

## 2 Model assumptions and mathematical representations

In this study, the flow of a nanofluid produced by stretching of an inclined cylindrical solid is considered. The flow involves the transfer of momentum, heat and nanoparticle in the vicinity of the solid surface. Using the conservation of thermal energy, mass and linear momentum, nonlinear differential equations are formulated followed by stating the boundary constraints. It is considered that the flow of the nanofluid is produced by the stretching of the cylinder. Such flow systems are encountered in material processing like wire drawing, fiber production and coating of metallic stripes.

**Figure 1:**
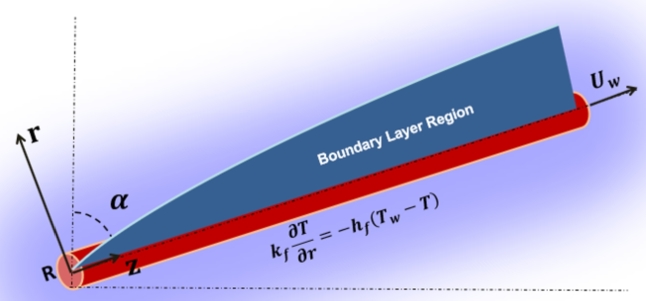
Schematic view of the flow problem.

Due to the curved nature of the surface, the cylindrical coordinates (z,r,θ) are used for which *z* and *r* correspond to the axial and normal directions respectively as outlined in Fig. 1.

The mass conservation principle for the flow configuration is given by the equation of continuity as follows [24], [25]:

(1) $$\frac{\partial u}{\partial z}+\frac{\partial w}{\partial r}+\frac{w}{r}=0$$

where *u* and *w* are the velocity decomposition in the axial (*z*) and the radial (*r*) directions of the cylinder, respectively.

Due to the electrical conductivity of the nanofluid, a constant magnetic force is applied along the transverse direction to manage the flow behavior from outside. Taking the impacts of buoyancy forces, angle of elevation and medium porosity, the conservation of momentum in cylindrical coordinates takes the form [24], [25]

(2) $$u\frac{\partial u}{\partial z}+w\frac{\partial u}{\partial r}=\upsilon \left(\frac{\partial^{2}u}{\partial r^{2}}+\frac{1}{r}\frac{\partial u}{\partial r}\right)-\left(\frac{\upsilon }{K_{0}}+\frac{\sigma B_{0}^{2}}{\rho }\right)u+g\left[\beta_{T}(T-T_{\infty })+\beta_{c}(C-C_{\infty })\right]sin(\alpha )$$

where the symbols *α*, $K_{0}$ and *B* define the angle that the cylinder makes from the vertical, coefficient of medium porosity and uniform magnetic field respectively; *g* is the gravitational force; the variables *ρ*, *σ* and *υ* correspond to density, electric conductivity and kinematic viscosity of the fluid; $\beta_{c}$ and $\beta_{T}$ are coefficients of volumetric concentration and thermal expansion; the pairs $\left(T,T_{\infty }\right)$ and $\left(C,C_{\infty }\right)$ correspond to temperature and nanoparticle concentration in the boundary layer and inviscid regions.

Since there are uniformly dispersed nanoparticles in the flow system, the conservation of thermal energy takes the impacts of thermophoresis and Brownian effects in to account. Further, considering the significant roles of heat exchange through concentration gradient, thermal radiation, heat source, heat dissipation due to viscous force and Joule heating, the equation of heat in the boundary layer region is given as [24], [25]

$$u\frac{\partial T}{\partial z}+w\frac{\partial T}{\partial r}=\alpha_{f}\left(\frac{\partial^{2}T}{\partial r^{2}}+\frac{1}{r}\frac{\partial T}{\partial r}\right)+\tau \left[D_{B}\frac{\partial T}{\partial r}\frac{\partial C}{\partial r}+\frac{D_{T}}{T_{\infty }}{\left(\frac{\partial T}{\partial r}\right)}^{2}\right]+\frac{16\sigma^{⁎}T_{\infty }^{3}}{3kk^{⁎}}\left(\frac{\partial^{2}T}{\partial r^{2}}+\frac{1}{r}\frac{\partial T}{\partial r}\right)$$

(3) $$+\frac{\sigma B_{0}^{2}}{\rho c_{p}}u^{2}+\frac{\upsilon }{c_{p}}{\left(\frac{\partial u}{\partial r}\right)}^{2}+\frac{D_{m}K_{T}}{C_{s}C_{p}}\frac{\partial^{2}C}{\partial r^{2}}+\frac{Q_{0}}{\rho c_{p}}(T-T_{\infty })$$

where the expression $\tau =\frac{{\left(\rho c_{p}\right)}_{p}}{{\left(\rho c_{p}\right)}_{f}}$ quantifies the ratio of heat capacities of nanoparticle to that of the base fluid with $c_{p}$ representing the specific heat capacity at constant pressure. The term $\alpha_{f}=\frac{k}{{\left(\rho C_{p}\right)}_{f}}$ is used to quantify the coefficient of thermal diffusivity. The variables *k*, $k^{⁎}$, $D_{m}$, $C_{s}$, $\sigma^{⁎}$, $K_{T}$ and $Q_{0}$ denote thermal conductivity of the fluid, Stefan-Boltzmann constant, species diffusivity, concentration susceptibility, mean thermal absorption coefficient, thermal diffusion ratio and coefficient of heat source respectively.

Taking the impacts of chemical reaction and mass transfer due to temperature difference, the conservation of nanoparticle volume fraction is governed by the following equation [24], [25]

(4) $$u\frac{\partial C}{\partial z}+w\frac{\partial C}{\partial r}=D_{B}\left[\frac{\partial^{2}C}{\partial r^{2}}+\frac{1}{r}\frac{\partial C}{\partial r}\right]+\frac{D_{T}}{T_{\infty }}\left[\frac{\partial^{2}T}{\partial r^{2}}+\frac{1}{r}\frac{\partial T}{\partial r}\right]+\frac{D_{m}K_{T}}{T_{m}}\frac{\partial^{2}T}{\partial r^{2}}-K_{r}(C-C_{\infty })$$

where $K_{r}$ and $T_{m}$ refer to the coefficient of chemical reaction and mean temperature.

On the other hand, the boundary conditions relevant to the flow problem are stated as follows. It is assumed that the cylindrical surface is stretched at a velocity of $U_{w}=\frac{U_{0}z}{L}$ where *L* and $U_{0}$ are constants for the reference length and velocity. An assumption is also made on the influence of velocity slip effect at the solid-fluid interface. It is also considered that the exchange of heat among the cylindrical surface and the surrounding fluid occurs in convective mechanism. Moreover, the nanoparticle concentration at the interface between the fluid and the cylinder is assumed to be constant. With these assumptions, the following additional conditions are developed at (r=R) where *R* is the radius of the cylinder [24], [25]

(5) $$u=U_{w}(z,t)+U_{slip},\;\;\;\;\;\;\;\;\;\;\;\;w=0,\;\;\;\;\;\;\;\;\;\;\;\;\;-k\frac{\partial T}{\partial r}=h_{f}(T_{w}-T),\;\;\;\;\;\;\;\;\;\;\;\;C=C_{w},$$

where $U_{slip}=\upsilon \frac{\partial u}{\partial r}N_{s}$ is the velocity slip condition with $N_{s}$ denoting the hydrolic slip effect; $h_{f}$ is the coefficient of convective heat transfer and $C_{w}$ is the constant value of nanoparticle concentration at the surface of the cylinder.

Again, outside the boundary layer region (r→∞), the temperature distribution, fluid velocity and concentration of nanoparticle are taken to be constant as given in [24], [25]

(6) $$u\to 0,\;\;\;\;\;\;\;\;\;\;T\to T_{\infty },\;\;\;\;\;\;\;\;\;\;and\;\;\;\;\;\;\;\;C\to C_{\infty }.$$

Further, the three useful events in the fluid flow phenomena near the solid surface are measured in terms of the quantities named by the local skin friction coefficient $C_{f}$, Nusselt number $Nu_{x}$ and Sherwood number $Sh_{x}$ which are given as [24], [25]

(7) $$C_{f}=\frac{\tau_{w}}{\rho_{f}U_{w}^{2}},\;\;\;\;\;\;\;\;\;Nu_{z}=\frac{zq_{w}}{\kappa (T_{w}-T_{\infty })},\;\;\;\;\;\;\;\;\;and\;\;\;\;\;\;Sh_{z}=\frac{zq_{m}}{D_{B}(C_{w}-C_{\infty })}$$

where the shear stress, heat and mass fluxes at the solid-fluid interface are given as [24], [25]

(8) $$\tau_{w}=\mu {\left[\frac{\partial u}{\partial r}\right]}_{r=R}^{2},\;\;\;\;\;\;\;\;q_{w}=-\left(\kappa +\frac{16\sigma^{⁎}T_{\infty }^{3}}{3k^{⁎}}\right){\left[\frac{\partial T}{\partial r}\right]}_{r=R},\;\;\;\;and\;\;\;\;q_{m}=-D_{m}{\left[\frac{\partial C}{\partial r}\right]}_{r=R}$$

## 3 Method of the study

The optimal homotopy analysis method (OHAM), first introduced by Liao, is an outstanding method of analysis that gives convenient mechanisms for ensuring the convergence of the solutions to a number of linear or nonlinear models that arise in science and engineering [26], [27]. In implementing the optimal homotopy analysis method to the strongly nonlinear models in Eqs. (1)-(7), the following efforts are made to make the established models convenient for computation. This involves nondimensionalizing the unknown functions and then changing the dimensional set of partial differential equations in to a more compact forms of ordinary differential equations. Also, the corresponding auxiliary quantities for the homotopy approximations are carefully selected and the whole algorithm is coded in Mathematica software.

To this end, a new variable

(9) $$\eta =\frac{1}{2}\sqrt{\frac{U_{o}}{\upsilon L}}\left(\frac{r^{2}-R^{2}}{R}\right)$$

is introduced [28], [29] that combines the cylindrical coordinates in to one. Next, the function

(10) $$\psi (r,z)=\sqrt{\frac{\upsilon U_{0}}{L}}zRf(\eta ),$$

is defined [28], [29] with the property that

(11) $$u=\frac{1}{r}\frac{\partial \psi }{\partial r}\;\;\;\;\;\;and\;\;\;\;\;\;w=-\frac{1}{r}\frac{\partial \psi }{\partial z}.$$

Here, f(η) is a dimensionless stream function. Consequently, the components of velocity are re-written as [28], [29]

(12) $$u=\frac{U_{0}z}{L}f^{\prime }(\eta )\;\;\;\;\;\;and\;\;\;\;\;\;w=-\frac{R}{r}\sqrt{\frac{\upsilon U_{o}}{L}}f(\eta ),$$

where $f^{\prime }(\eta )$ is the dimensionless velocity profile.

Finally, the temperature and concentration distributions are made dimensionless by defining new functions as [28], [30]

(13) $$\theta (\eta )=\frac{T-T_{\infty }}{T_{w}-T_{\infty }}$$

and

(14) $$\varphi (\eta )=\frac{C-C_{\infty }}{C_{w}-C_{\infty }}.$$

Now, deriving the essential derivatives of the dimensionless variable and functions and then replacing them in the governing equations, boundary conditions and the other quantities of interest, one can check that the equation of continuity in Eq. (1) is satisfied identically. On the other hand, the partial differential equation for the conservation of momentum in Eq. (2) is condensed to an ordinary differential equation of the form [29], [31]

(15) $$(1+2K\eta )f^{\prime \prime \prime }+2Kf^{\prime \prime }+ff^{\prime \prime }-f^{\prime \;2}-(K_{p}+M)f^{\prime }+[G_{r}\theta +G_{m}\varphi ]sin(\alpha )=0,$$

where the prime(s) ′ define the derivative of the unknown function in relation to *η*. The parameters $K=\sqrt{\frac{\upsilon L}{U_{0}R^{2}}}$, $Kp=\frac{\upsilon z}{K_{0}U_{w}}$ and $M=\sqrt{\frac{\sigma B_{0}^{2}L}{\rho U_{0}}}$ correspond to the curvature of the cylindrical surface, porosity of the medium and magnetic field intensity. The expressions $G_{r}=\frac{g\beta_{T}(T_{w}-T_{\infty })L^{2}}{zU_{0}^{2}}$ and $G_{m}=\frac{g\beta_{c}(C_{w}-C_{\infty })L^{2}}{zU_{0}^{2}}$ are thermal and mass Grashof numbers used to quantify the effects of buoyancy forces.

The simplification process also helps to reduce the conservation of thermal energy in Eq. (3) to the form [29], [31]

$$\frac{1}{Pr}\left(1+\frac{4}{3}Rd\right)[(1+2K\eta )\theta^{\prime \prime }+2K\theta^{\prime }]+f\theta^{\prime }+(1+2K\eta )\left[N_{b}\theta^{\prime }\varphi^{\prime }+N_{t}\theta^{\prime \;2}\right]$$

(16) $$+Ec\left[(1+2K\eta )f^{\prime \prime \;2}-Mf^{\prime \;2}\right]+D_{f}\varphi^{\prime \prime }+Q\theta =0,$$

where the parameters $P_{r}=\frac{\upsilon }{\alpha_{f}}$, $Rd=\frac{4\sigma^{⁎}T_{\infty }^{3}}{3kk^{⁎}}$, $N_{b}=\frac{\tau D_{B}(C_{w}-C_{\infty })}{\upsilon }$, $N_{t}=\frac{\tau D_{T}(T_{f}-T_{\infty })}{\upsilon T_{\infty }}$, $D_{f}=\frac{D_{m}K_{T}}{\upsilon C_{s}C_{p}}\frac{C_{w}-C_{\infty }}{T_{w}-T_{\infty }}$, $Ec=\frac{U_{w}^{2}}{{\left(C_{p}\right)}_{f}(T_{w}-T_{\infty })}$ and $Q=\frac{zQ_{0}}{{\left(\rho C_{p}\right)}_{f}U_{w}}$ correspond to the Prandtl number, thermal radiation, Brownian motion, thermophoresis property, Eckert number, Dufour number and heat generation or absorption effects respectively.

Again, the conservation of nanoparticle volume fraction defined in Eq. (4) is simplified to the form [29], [31]

(17) $$(1+2k\eta )\varphi^{\prime \prime }+2k\varphi^{\prime }+\frac{N_{t}}{N_{b}}[(1+2k\eta )\theta^{\prime \prime }+2k\theta^{\prime }]+Sc(f\varphi^{\prime }+S_{r}\theta^{\prime \prime }-\gamma \varphi )=0,$$

where the parameters $Sc=\frac{\upsilon }{D_{B}}$, $S_{r}=\frac{D_{m}K_{T}}{T_{m}\upsilon }\frac{T_{w}-T_{\infty }}{C_{w}-C_{\infty }}$ and $\gamma =\frac{K_{r}z}{U_{w}}$ stand for the Schmidt number, Soret number and chemical reaction effects respectively.

Similarly, the boundary conditions in Eqs. (5)-(6) are simplified to the following expressions [29], [31]

(18) $$f(\eta )=0,\;\;\;f^{\prime }(\eta )=1+N_{f}f^{\prime \prime }(0),\;\;\;\theta^{\prime }(\eta )=-Bi[1-\theta (\eta )],\;\;\;\varphi (\eta )=1\;\;\;for\;\;\;\eta =0$$

and

(19) $$f^{\prime }(\eta )\to 0,\;\;\;\theta (\eta )\to 0,\;\;\;\varphi (\eta )\to 0\;\;\;as\;\;\;\eta \to \infty ,$$

where the parameter $N_{f}=N_{s}\upsilon Re^{1/2}z^{-1/4}$ defines the momentum slip with $Re=\frac{U_{w}z}{\upsilon }$ denoting the Reynolds number. The expression $Bi=\frac{h_{f}}{k}\sqrt{\frac{\upsilon L}{U_{w}}}$ stands for the Biot number for convective heat transfer.

Further, the local numbers for the fluid transport rates given in Eq. (7) are described in a more condensed form as [29], [31]

(20) $$C_{f}=Re_{z}^{-1/2}f^{\prime \prime }(0),\;\;\;Nu_{z}=-Re_{z}^{1/2}\left(1+\frac{4}{3}R_{d}\right)\theta^{\prime }(0)\;\;\;and\;\;\;Sh_{z}=-Re_{z}^{1/2}\varphi^{\prime }(0)$$

At this point it is worth mentioning that the derivatives $f^{\prime \prime }(0)$, $\theta^{\prime }(0)$ and $\varphi^{\prime }(0)$ at the solid surface are important quantities to give equivalent estimates for the local skin friction coefficient, Nusselt number and Sherwood number that actually correspond to the rate of change of momentum, heat transfer and concentration diffusion around the solid surface.

Now, the homotopy approximations for the dimensionless functions given in Eqs. (15)-(17) along with the simplified expressions of the boundary conditions in Eqs. (18)-(19) can be determined at any point across the region. To this end, the initial values of the unknown functions are defined in relation to the identified boundary conditions as follows [31], [32]

(21) $$f_{0}(\eta )=\frac{1-e^{-\eta }}{1+N_{f}},\;\;\;\;\;\;\;\;\;\theta_{0}(\eta )=\frac{Bi}{1+Bi}e^{-\eta }\;\;\;\;\;\;\;\;\;and\;\;\;\;\;\;\varphi_{0}(\eta )=e^{-\eta }.$$

Then by continuous mapping of the homotopy functions, the initial estimations of the unknown functions are mapped to the actual values of the unknown functions [26]. Again, the corresponding linear operators are formulated as [26]

(22) $$L_{f}=f^{\prime \prime \prime }+f^{\prime \prime },\;\;\;\;\;\;L_{\theta }=\theta^{\prime \prime }+\theta^{\prime }\;\;\;\;\;\;and\;\;\;\;\;\;L_{\varphi }=\varphi^{\prime \prime }+\varphi^{\prime }$$

From the qualitative understanding on the nature of the flow configuration, it appears to be reasonable in choosing the auxiliary functions for the homotopy approximations as [26]

(23) $$H_{f}(\eta )=H_{\theta }(\eta )=H_{\varphi }(\eta )=e^{-\eta }.$$

One of the unique features of the homotopy analysis method is its ability to provide convenient ways of ensuring convergence of analytic approximations. With the proper selection of the convergence control parameters $\left(\hbar_{i}\right)$, one can regulate the rate of convergence of the approximated solutions. The most efficient values of $\hbar_{i}$ for the functions under consideration are identified by reducing the following residual errors [26]

(24) $$\varepsilon_{k}^{i}(\hbar_{i})\approx \frac{1}{N+1}\sum_{j=0}^{N}{\left\{{ℵ}_{i}\left[\sum_{n=0}^{k}\phi_{n}^{i}(\eta )\right]\right\}}^{2}$$

where $\phi^{i}$ and ${ℵ}_{i}$ are the homotopy approximation for the unknown functions and the corresponding nonlinear operators.

The values of parameters $Sc=2,Pr=5,K=Nt=0.1,Nb=0.2,M=1,Kp=0.1;B_{i}=0.5,Gm=0.2,Gr=0.3,Rd=0.1,Ec=0.01,Q=0.1,\gamma =0.2,\alpha =\pi /6,Df=0.01,Sr=0.02,Nf=0.1$ are used through out the manuscript except for the parameter being studied. Now, using the Mathematica symbolic software, the method has been implemented successfully to obtain the necessary graphical or numerical outputs. The first important activity in this symbolic computation gives $\hbar_{f}\approx -0.511,\;\;\hbar_{\theta }\approx -4.529$ and $\hbar_{\varphi }\approx -0.799$, as the optimal values for the convergence control parameters so that the residual errors $\varepsilon_{f}$, $\varepsilon_{\theta }$ and $\varepsilon_{\varphi }$ are minimized for increasing orders of approximations as shown in Table 1.

**Table 1:** Residual errors with order of approximations.

Order of approx	Average Squared Residual Errors		
	*ε*_*f*_	*ε*_*θ*_	*ε*_*φ*_
2	1.67 × 10^−5^	2.13 × 10^−6^	3.52 × 10^−7^
6	1.12 × 10^−6^	1.19 × 10^−7^	2.25 × 10^−7^
10	2.97 × 10^−7^	8.01 × 10^−9^	1.19 × 10^−7^
14	1.12 × 10^−7^	6.13 × 10^−10^	7.65 × 10^−8^
18	5.26 × 10^−8^	7.68 × 10^−11^	5.62 × 10^−8^
22	3.09 × 10^−8^	2.23 × 10^−11^	4.22 × 10^−8^
26	2.19 × 10^−8^	1.16 × 10^−11^	3.19 × 10^−8^
30	1.75 × 10^−8^	7.40 × 10^−12^	2.43 × 10^−8^

**Figure 2:**
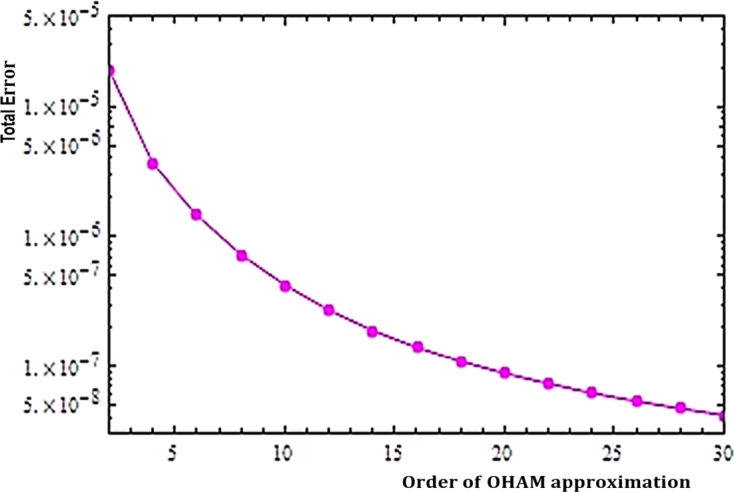
Total Residual Errors in relation to the HAM Approximations.

In a more systematic presentation, the features of the residual errors are combined to give a total residual error as sketched in Fig. 2. It can easily be identified that the total residual error decays for higher orders of the approximation. This decreasing error due to the proper choice of the convergence control parameters ensures the accuracy of the approximated analytic solutions.

Further, the convergence of the approximated solutions is investigated by computing the values of some relevant quantities of interest at different orders of approximation. Table 2 displays the values of the essential derivatives $-f^{\prime \prime }(0)$, $-\theta^{\prime }(0)$ and $-\varphi^{\prime }(0)$ against the order of approximations.

**Table 2:** Computed values of the essential derivatives with order of approximations.

Order of approximations	−*f*″(0)	−*θ*′(0)	−*φ*′(0)
2	1.12015	0.26233	1.01707
6	1.14513	0.25871	1.03742
10	1.14552	0.26124	1.03268
14	1.14566	0.26216	1.03098
18	1.14573	0.26241	1.03067
22	1.14577	0.26246	1.03072
26	1.14580	0.26247	1.03083
30	1.14581	0.26247	1.03093

Table 2 outlines that the computed values of interest are essentially very close to each other as the order of approximation increases. This shows that the solution error is very small and the approximated solutions for the unknown functions are convergent.

Since the present mathematical model is a generalization of some existing models and that the method is implemented to this problem for the first time, computations are made to compare the findings of the present work with that of previous reports under similar considerations. To this end, the early works Murthy et al. [28] and Fang et al. [33] are considered for analyzing the validity of the present work (Table 3).

**Table 3:** Different computational values of −*f*″(0) when *Bi* = 0.5, *Nt* = 0.1, *Nb* = 0.2, *Sc* = 11, *Pr* = 10, *K* = *K*_*p*_ = *Gc* = *Gr* = *D*_*f*_ = *α* = *Rd* = *S*_*r*_ = *Ec* = *N*_*f*_ = *Q* = *γ* = 0 for selected values of the magnetic field parameter.

*M*	−*f*″(0) [33]	−*f*″(0) [28]	Present Study		
			−*f*″(0)	−*θ*′(0)	*φ*′(0)
0.0	-	1.0000	1.000000	1.077323	0.528491
0.2	-	1.0198	1.019744	1.110775	0.543299
0.5	1.1180	1.1180	1.117860	1.251178	0.614545
0.8	-	1.2606	1.280537	1.369809	0.680782
1.0	-	1.4142	1.414187	1.385146	0.692252

The computed values of $-f^{\prime \prime }(0)$ for the current work are in a good agreement to that of the early works as tabulated in Table 3. Also, important results on the values of $-\theta^{\prime }(0)$ and $\phi^{\prime }(0)$ are included in Table 3.

With all these reasonable convergence, accuracy and validity of the proposed method and flow model, detail parametric analysis follows for the intended fluid transport rates in the results and discussion part of this report.

## 4 Results and discussion

The impacts of significant parameters on the fluid transport rates are presented graphically against the smooth variation of parameters. To this end, the nature of the derivatives $f^{\prime \prime }(0),\theta^{\prime }(0)$ and $\phi^{\prime }(0)$ are examined in the continuous domains of the parameters. The reliability of the mathematical simulation is further supported by the realities associated to the nature of the quantities in response to the parameters.

### 4.1 Effects of angle of inclination

**Figure 3:**
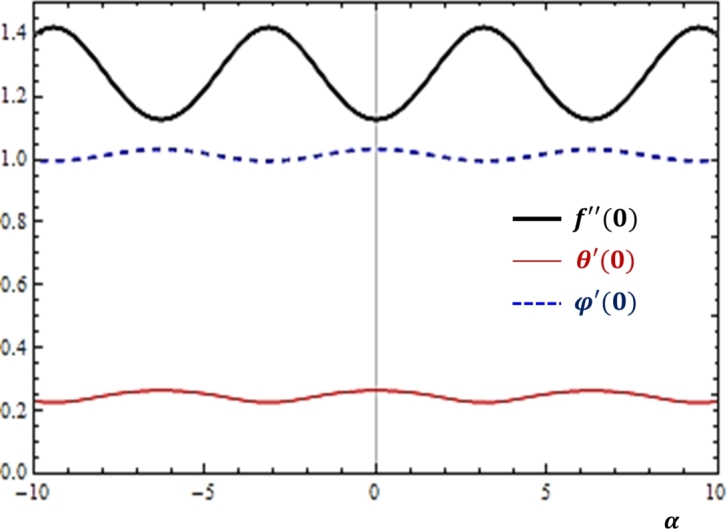
Effects of angle (*α*) on *f*″(0), *θ*′(0) and *ϕ*′(0).

The effect of angle (*α*) on the fluid transport rates are studied in the present study as shown in Fig. 3. It can be noted from Fig. 3 that as the angle (*α*) changes, a more significant variation of momentum transfer rate is noticed and less variations are detected for the concentration and heat fluxes. This corresponds to the fact that changing the angle of inclination is mainly responsible for changing the effect of gravity which in turn has the tendency to influence the flow momentum than any other phenomena.

### 4.2 Effects of medium porosity

**Figure 4:**
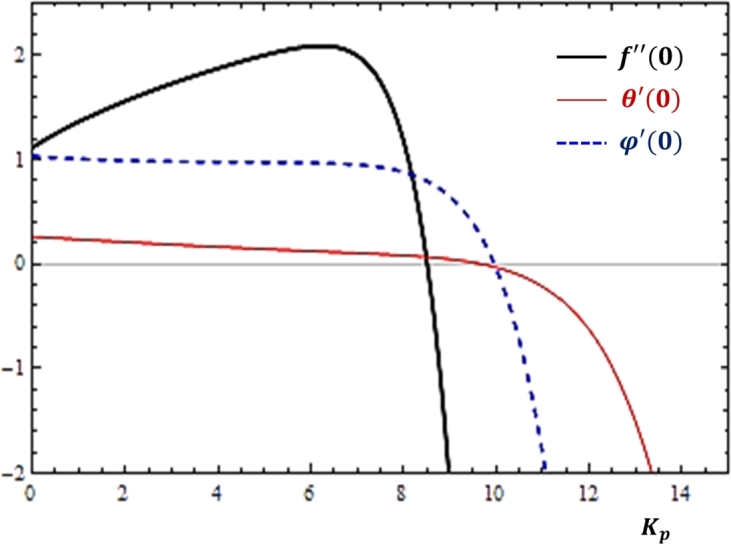
Effects of medium porosity (*K*_*p*_) on *f*″(0), *θ*′(0) and *ϕ*′(0).

There are several forms of fluid flows that occur through medium filled with pores. Understanding the effects of medium porosity is thus helpful to improve the working efficiency of many industrial and engineering activities. In this study, the impact of the porosity parameter on the fluid transport rates is presented in Fig. 4. Fig. 4 reveals that by increasing the values of the porosity parameter $K_{p}$, the three rates of fluid transport phenomena can be very low. This is evident from the definition that greater values of $K_{p}$ correspond to minimum permeability nature of the medium that actually restricts the fluid transport rates as expected.

### 4.3 Magnetic field effects

**Figure 5:**
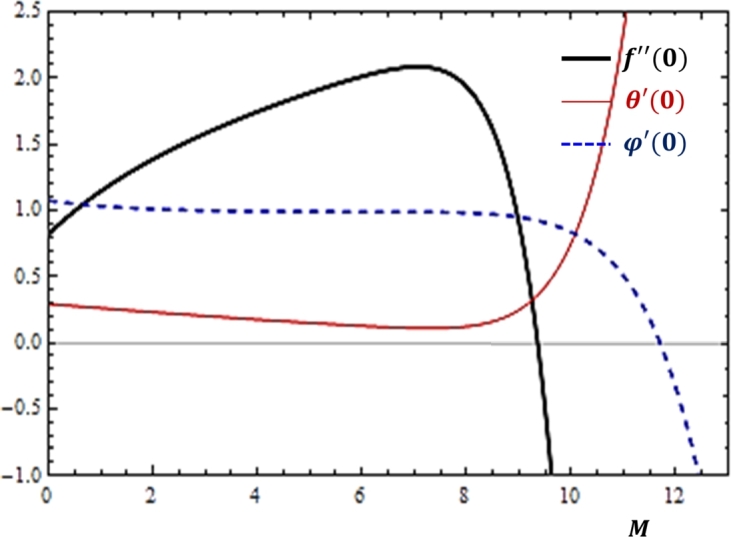
Effects of Magnetic field (*M*) on *f*″(0), *θ*′(0) and *ϕ*′(0).

Due to the electrical conductivity of the nanofluid considered in the flow problem, it is worth examining how the fluid transport rates are changing with the increase in magnetic field parameter. Fig. 5 is sketched to portray the nature of the quantities associated to the three rates of fluid transport in relation to the variation of the parameter. According to the results shown in Fig. 5, for smaller values of M, an improvement on momentum transfer rate is noticed while no considerable variation is exhibited by the heat and mass transfer rates. However, for larger values of M, a significant variation in all the transport rates is observed. Particularly, it is shown that as the magnetic field intensity increases, the heat flux grows rapidly while the concentration and momentum diffusion rates are slowed down. This observation also holds true from the practical point of view. That is, the applied magnetic field generates an electromagnetic force against the direction of fluid flow and it produces heat due to the work done against the effect of magnetic field.

### 4.4 Effects of buoyancy forces

**Figure 6:**
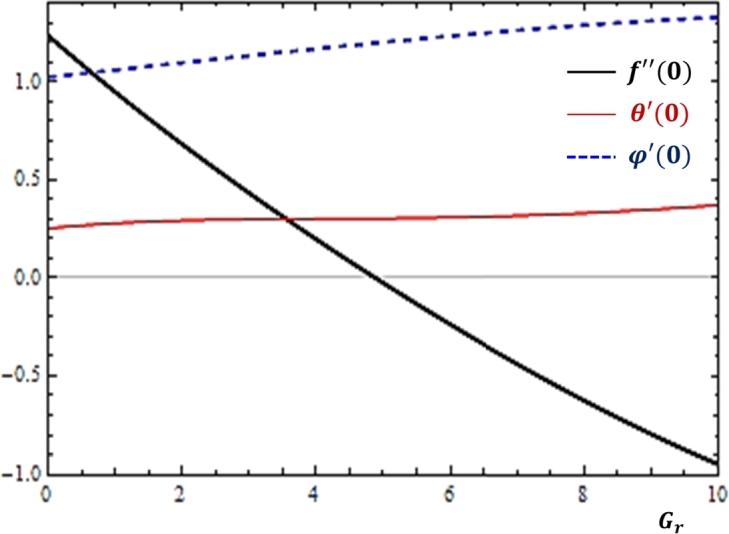
Impacts of thermal Grashof number (*G*_*r*_) on *f*″(0), *θ*′(0) and *ϕ*′(0).

**Figure 7:**
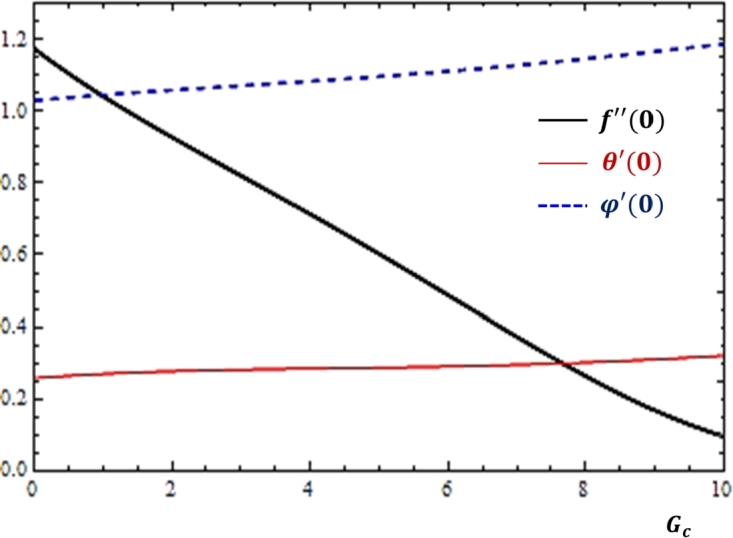
Impacts of mass Grashof number (*G*_*c*_) on *f*″(0), *θ*′(0) and *ϕ*′(0).

The thermal and concentration buoyancy forces are body forces that are produced as a result of temperature and concentration differences respectively. The quantitative analysis of these forces is made with the help of the Grashof numbers. Figure 6, Figure 7 are plotted so as to disclose the features of the quantities related to fluid transport rates with the variations in the Grashof numbers. Based on the results displayed in Figure 6, Figure 7, as the values of the Grashof numbers increase, momentum transfer is declined while mass and heat transfers show no considerable variation with the parameters. Here, it is important to observe that at any value of the Grashof numbers, the mass transfer rate is greater than the heat transfer rate.

### 4.5 Cross diffusion effects

**Figure 8:**
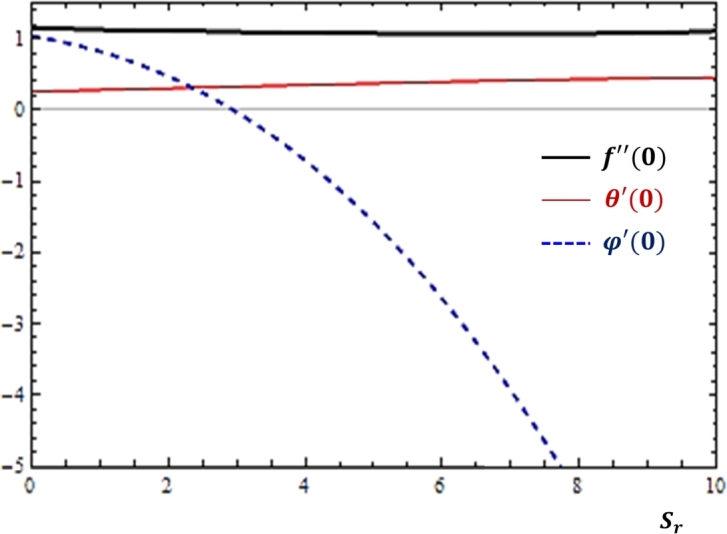
Effects of Soret number (*S*_*r*_) on *f*″(0), *θ*′(0) and *ϕ*′(0).

**Figure 9:**
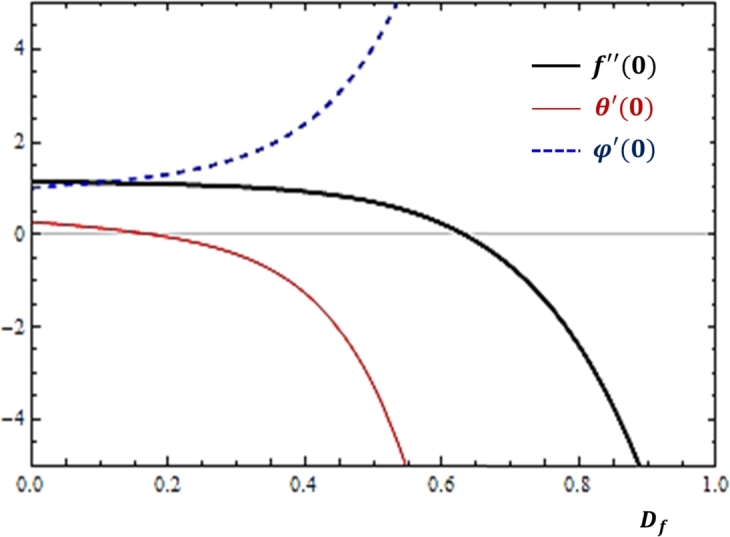
Effects of Dufour number (*D*_*f*_) on *f*″(0), *θ*′(0) and *ϕ*′(0).

The Soret and Dufour numbers are used to analyze the development of mass transfer due to temperature gradient and heat transfer as a result of concentration difference. The nature of fluid transport rates near the cylinder with respect to these cross diffusion effects is studied for the current problem and the findings are reported as shown in Figure 8, Figure 9. As depicted in Figure 8, Figure 9 that the increase in the Soret number slows down the rate of mass diffusion while mass and momentum transfer rates do not change with the parameter. However, in Fig. 9, the mass transfer rate grows while heat and momentum transfer rates are slowed down with the increase in the Dufour number.

### 4.6 Thermal radiation effects

**Figure 10:**
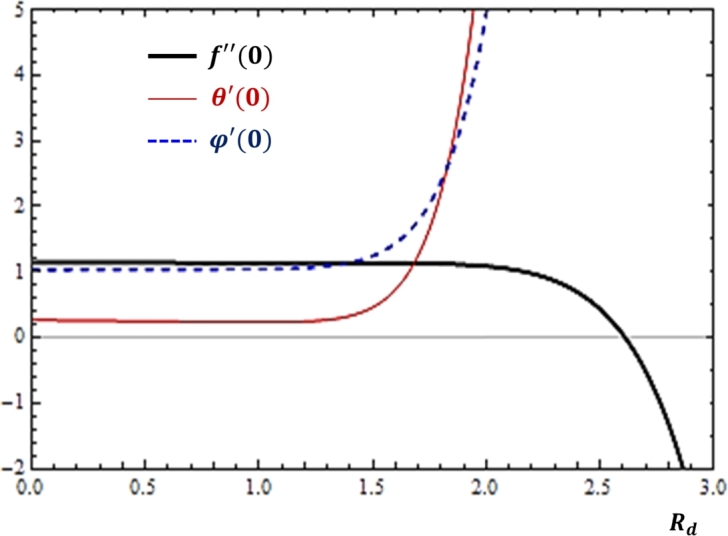
Impacts of thermal radiation (*Rd*) on *f*″(0), *θ*′(0) and *ϕ*′(0).

Considering that the flow phenomena are made to happen in situations where there is high temperature, the role of thermal radiation in influencing the rates of fluid transport has been studied and as shown in Fig. 10. It is found that for greater values of the thermal radiation, both heat transfer and mass transfer rates are boosted while momentum transfer rate is depreciated as depicted in Fig. 10.

### 4.7 Effects of heat generation or absorption

**Figure 11:**
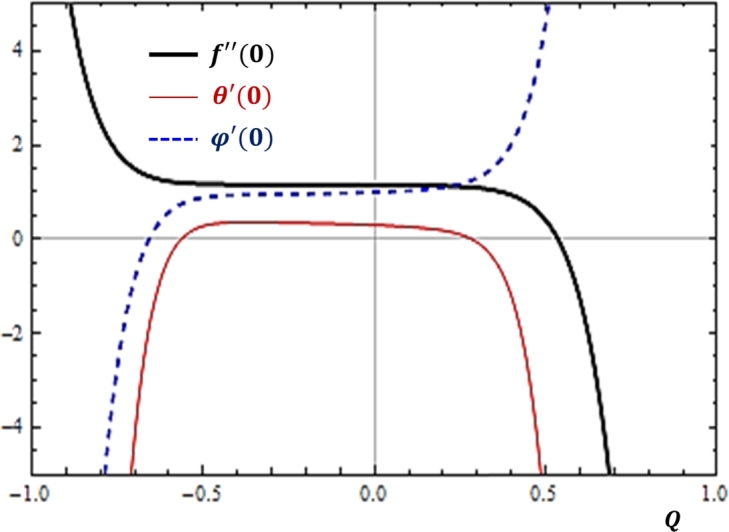
Effects of heat absorption (*Q* < 0) and heat generation (*Q* > 0) on *f*″(0), *θ*′(0) and *ϕ*′(0).

It is shown in Fig. 11 that for larger values of heat absorption (Q>0) parameter, the mass transfer rate is enhanced while momentum and heat transfer rates are depreciated. On the other hand, for larger values of heat generation (Q<0) parameter, it is found that the momentum transfer rate is enhanced while concentration and heat transfer rates are depreciated.

## 5 Conclusions

The investigation on rates of heat, mass and momentum transfer in an electrically conducting nanofluid over a cylindrical solid is made in the presence of several effects. The transport phenomena of the nanofluid are described mathematically by using partial differential equations. To effectively manage the computation, suitable variables are introduced and the partial differential equations are reduced to ordinary differential forms. Then the powerful optimal homotopy analysis method has been successfully applied to examine to obtain analytic approximations for the unknown quantities. Upon the analysis, the following major observations are noted:

• The heat transfer near the solid surface can be facilitated by adding the effect of magnetic field or thermal radiation. However, it is found that increasing the effects of medium porosity, Dufour number, heat generation or absorption slow down the heat transfer rate.
• It can be concluded that mass transfer rate can be enhanced with the increase in thermal radiation, heat generation or Dufour effects. A slight improvement in mass transfer rate is also noticed with the growth in the buoyancy forces. On the other hand, mass transfer rate declines rapidly with the rises in Soret effect, heat absorption, porosity or magnetic field parameter.
• Rates of momentum transfer around the solid can be accelerated by introducing small effects of magnetic field or porosity in the medium. In contrast, momentum transfer decelerates by inducing the effects of buoyancy forces, Dufour effect, heat generation or thermal radiation. Further, a considerable variation with the rates of momentum diffusivity is noticed for a range of angle of elevations. However, the effect of Soret number shows no direct impact on momentum transfer rate.

## Funding

No specific fund is allocated for this work.

## CRediT authorship contribution statement

**Tadesse Walelign:** Writing – review & editing, Writing – original draft, Visualization, Validation, Software, Project administration, Methodology, Investigation, Formal analysis, Conceptualization.

## Declaration of Competing Interest

The authors declare the following financial interests/personal relationships which may be considered as potential competing interests: Tadesse Walelign reports administrative support was provided by 10.13039/501100022150Debre Tabor University. If there are other authors, they declare that they have no known competing financial interests or personal relationships that could have appeared to influence the work reported in this paper.

## Data Availability

No data were used to support this study.
